# A Hierarchical Learning Approach for Human Action Recognition

**DOI:** 10.3390/s20174946

**Published:** 2020-09-01

**Authors:** Nicolas Lemieux, Rita Noumeir

**Affiliations:** Electrical Engineering Department, École de Technologies Supérieure, Montreal, QC H3C 1K3, Canada; Rita.Noumeir@etsmtl.ca

**Keywords:** human action recognition, HAR, explainable deep-learning, 1D CNN, one-vs.-all, high-level activity recognition, intelligent sensors, modular method, light-weight method

## Abstract

In the domain of human action recognition, existing works mainly focus on using RGB, depth, skeleton and infrared data for analysis. While these methods have the benefit of being non-invasive, they can only be used within limited setups, are prone to issues such as occlusion and often need substantial computational resources. In this work, we address human action recognition through inertial sensor signals, which have a vast quantity of practical applications in fields such as sports analysis and human-machine interfaces. For that purpose, we propose a new learning framework built around a 1D-CNN architecture, which we validated by achieving very competitive results on the publicly available UTD-MHAD dataset. Moreover, the proposed method provides some answers to two of the greatest challenges currently faced by action recognition algorithms, which are (1) the recognition of high-level activities and (2) the reduction of their computational cost in order to make them accessible to embedded devices. Finally, this paper also investigates the tractability of the features throughout the proposed framework, both in time and duration, as we believe it could play an important role in future works in order to make the solution more intelligible, hardware-friendly and accurate.

## 1. Introduction

As human beings, part of our happiness or suffering depends on our interactions with our direct environment, which is governed by an implicit or explicit set of rules [1]. Meanwhile, a growing proportion of these interactions are now targeted towards connected devices such as smart phones, smart watches, intelligent home assistants and other IoT objects since their number and range of applications keep growing every year [2]. As a consequence of this phenomenon, efforts are made in order to improve the users’ experience through the elaboration of some ever-more intuitive and comfortable human-machine interfaces [3]. With the relatively recent successes of artificial intelligence based algorithms and the emergence of a wide range of new sensors, human action recognition (HAR) has become one of the most popular problems in the field of pattern recognition currently and plays a key role in the development of such interfaces. In that context, RGB, depth, skeleton and infrared stream based approaches attracted most of the scientific community’s attention as their non-invasive nature is well suited for applications such as surveillance and led to the creation of commonly accepted benchmarks such as NTU-RGB-D [4], which was recently enhanced as NTU-RGB-D 120 [5]. On the other hand, action recognition based on visual streams can only be used within limited setups, is prone to issues such as occlusion and often needs substantial computational resources.

Admittedly less popular, HAR based on inertial sensor signals is still a relevant subject of research. Despite the fact that with this approach, the users are required to wear some kind of device, thanks to the miniaturization of microelectromechanical systems (MEMS), inertial measurement units (IMU) are now seamlessly integrated with different day-to-day objects such as remote controls or smart-phones and wearable technologies such as smart watches or smart cloths. Furthermore, for applications such as sports analysis, fall detection or remote physiotherapy, it provides an interesting alternative, as it can be used anywhere, provides more privacy and is considerably less expensive.

Although many different datasets exist in that branch of HAR, none have really established themselves as a clear benchmark, as placement strategies of the IMUs, or the set of activities are mostly targeted towards specific applications. In this work, we opted for the University of Dallas, Texas, multimodal human action dataset (UTD-MHAD) in order to validate our approach, as we believed that it offered the most variability in terms of activities (27) and actors (eight). Not only did the proposed method achieve competitive results on the classification task using IMU signals, it was also designed in order to address two of the biggest challenges currently faced by HAR algorithms [6]. For that purpose, we propose a hierarchical representation of the different actions by sampling the maximum and minimum activation of each convolution kernel throughout the network, which could potentially help with (1) the recognition of high-level activities characterized by the large time variations in the sub-movements composing them. Moreover, as the proposed solution is built around a 1D-CNN architecture, it was also observed that it necessitated dramatically less operations or memory than the most popular architectures, thus greatly contributing to (2) the portability to limited resource electronics for HAR algorithms. Finally, in this paper, we also investigated the tractability of the features throughout the proposed framework, both in time and duration, as we believe it could play an important role in future works in order to make the solution more intelligible, hardware-friendly, robust and accurate. For the rest of the present article, the following structure will observed:

- Section 2 quickly reviews the literature.
- Section 3 provides a detailed explanation of the method.
- Section 4 details the temporal analysis, showing how feature elements can be localized in time and that they report the dynamics of different durations.
- Section 5 provides the results in order to validate both the performances and properties of the proposed method.
- Section 6 summarizes the important results and their implications and provides ideas for future work.

## 2. Related Works

### 2.1. Neural Network Based HAR

Originally based on conventional machine learning methods such as support vector machines (SVM), decision trees or k-nearest neighbour (k-NN), early methods for HAR relied on various time and/or frequency domain features extracted from the raw data. However, as for many other fields, inertial sensor based HAR benefited from gradient descent based methods. Besides improving the classification capabilities, these methods also alleviated the requirement of feature engineering as they learn the features and classifier simultaneously from the annotated data. As a matter of fact, Kwapisz et al. [7] observed that multi-layer-perceptrons (MLPs) were superior to decision trees and logistic regression for HAR. Similarly, Weiss et al. [8] observed that MLP outperformed decision trees, k-NN, naive Bayes and logistic regression when the training and evaluation was done on data gathered from the same user and also noted that these user-specific models performed dramatically better than impersonal models where training and evaluation users are different. Since then, models based on convolution neural networks (CNN) have been proposed for inertial sensor based HAR [9,10,11,12] and have also been proven superior to k-NN and SVM [11]. With these methods, the input signals usually go through multiple convolution and pooling layers before being fed to a classifier. Although 1D-CNN architectures are well suited for the time series type of data such as IMU signals, CNNs are more famous in their 2D form, considering the tremendous amount of success they have had in the field of image recognition. Consequently, Jiang et al. [13] proposed to construct an activity image, based on IMU signals, in order to proceed to HAR as an image recognition problem. Back to 1D-CNN, Ronao et al. [12] showed that wider kernels and a low pooling size resulted in better performances. More recently, Murad et al. [14] implemented a recurrent neural network (RNN) and demonstrated the prevalence of their method over SVM and k-NN. Moreover, employing the long short-term memory (LSTM) variation of RNN along with CNNs, Hammerla et al. [15] and Ordonez et al. [16] achieved better results than with some strictly CNN based methods. However, the CNN architectures they used in their comparative study had respectively a maximum of three and four convolution blocks. Meanwhile, Imran and Raman [9] conducted an analysis on the topology of the 1D-CNN, from which they concluded that five consecutive blocks of convolution and pooling layers were better than four. Furthermore, with their architecture and a data augmentation strategy, they also achieved state-of-the-art results on the UTD-MHAD dataset for both sensor based and multi-modal HAR. Yet, leaving data augmentation aside, the proposed method in this article slightly outperformed theirs for the sensor based HAR.

### 2.2. Temporal Normalization

As the popular machine learning frameworks require the inputs to be of a constant shape during the training process and as different actions usually have different durations, temporal normalization is mandatory. On that account, different strategies have been proposed. One way to do it is by randomly sampling a fixed number of frames from a longer sequence while keeping the chronological ordering [9,15]. It can also serve as a data augmentation technique, since the sampled frames can vary from one epoch to another. Another way to do it is to sample a fixed amount of consecutive frames from the action sequence [8]. In this case, the algorithms often have to learn from incomplete representations. Finally, a more conservative way to achieve temporal normalization is to extend, up to a fixed point, the signals with zeros. This technique is also known as zero-padding. It has the benefit of enabling the algorithms to learn features on complete and undistorted sequences. Therefore, it will be the preferred strategy in this work. Moreover, contrary to Imran and Raman [9], who reported that the performances of their CNN based method decayed with the augmentation of the amount of zero-padding, our method proved itself to be robust against it.

## 3. Proposed Method

Like the majority of HAR algorithms, the main objective of our method is to provide a way to successfully classify specific actions based on a multivariate time series input. In the sensor based variation of HAR addressed here, the input time series, $X^{0}$, consist of a fixed number of time steps, $T_{0}$, each reporting a dynamic occurring at a certain moment *t*, by providing, in a vector form, $x_{t}$, the speed and angular rate variations, γ and ω, with respect to a 3-dimensional (x,y,z) orthogonal coordinate system whose origin corresponds to the IMU sensor. This can be expressed mathematically as:

(1) $$X^{0}=\left[x_{1}^{0},\ldots x_{t}^{0},\ldots x_{T_{0}}^{0}\right]\;where\;x_{t}^{0}=\left[\begin{array}{c} \gamma_{t}^{x}\\ \gamma_{t}^{y}\\ \gamma_{t}^{z}\\ \omega_{t}^{x}\\ \omega_{t}^{y}\\ \omega_{t}^{z} \end{array}\right]$$

In order to train our algorithm, the original time series, $X^{0}$, is conveyed to a 1D-CNN constituted of multiple convolution blocks in cascade (i.e., the output of a specific block serves as the input for the next), each applying a certain set of convolution filters, batch normalisation and max pooling. From this process, *L* other time series are created, ${\left\{X^{l}\in R^{F_{l}\times T_{l}}\right\}}_{l=1}^{L}$. Here, *L* is the number of convolution blocks; $F_{l}$ is the number of filters in the *l*th convolution block’s filter bank, $W^{l}\in R^{F_{l}\times m_{l}\times F_{l-1}}$ (whose filters are of size $m_{l}$ along the temporal axis); $T_{l}$ is the number of corresponding time steps of the *l*th generated time series, $X^{l}$. Hence, the *l*th time series is described as:

(2) $$X^{l}=\left[x_{1}^{l},\ldots x_{t}^{l},\ldots x_{T_{l}}^{l}\right]\;where\;x_{t}^{l}={\left[x_{t}^{l,1},\ldots x_{t}^{l,k},\ldots x_{t}^{l,F_{l}}\right]}^{T}$$

In these blocks, convolutions are carried without bias; thus, they can be described by the following equation:

(3) $$z_{t}^{l}=\phi \left(W^{l}*X_{\left[t,t+m_{l}-1\right]}^{l-1}\right)$$

where $z_{t}^{l}$ is the vector resulting from the application of all the convolution filters of $W^{l}$ to the portion of the previous time series whose vectors are between the *t*th and $\left(t+m_{l}-1\right)$th time steps inclusively, $X_{\left[t,t+m_{l}-1\right]}^{l-1}$; ϕ is the SeLU activation function [17], which demonstrated better experimental results; and ∗ is the convolution operator, which can be equivalently expressed as Equation (4) where $W_{i}^{l,k}$ is the *i*th column of the *k*th filter of $W^{l}$.

(4) $$W^{l,k}*X_{\left[t,t+m_{l}-1\right]}^{l-1}\equiv \sum_{i=1}^{m_{l}}\langle {\left(W_{i}^{l,k}\right)}^{T},x_{t+i-1}^{l-1}\rangle$$

Subsequently applying batch normalization and max pooling to the convolution output, the elements of the time step vector, $x_{t}^{l,k}$, of the different time series, ${\left\{X^{l}\right\}}_{l=1}^{L}$, are calculated as:

(5) $$x_{t}^{l,k}=\max\left\{BN_{\Gamma^{k},\beta^{k}}\left(z_{2t}^{l,k}\right),BN_{\Gamma^{k},\beta^{k}}\left(z_{2t+1}^{l,k}\right)\right\}$$

where $BN_{\Gamma^{k},\beta^{k}}\left(z_{2t}^{l,k}\right)$ is the batch normalization operation, which is defined as:

(6) $$BN_{\Gamma^{k},\beta^{k}}\left(z_{t}^{l,k}\right)=\frac{z_{t}^{l,k}-\mu_{B}^{k}}{\sqrt{{\left(\sigma_{B}^{k}\right)}^{2}+\epsilon }}\times \Gamma^{k}+\beta^{k}$$

where $\mu_{B}^{k}$ and ${\left(\sigma_{B}^{k}\right)}^{2}$ refer to the mean and variance of the elements of the *k*th dimension computed on the examples of the mini-batch, *B*; ϵ is an arbitrarily small constant used for numerical stability; and finally, Γ and β are parameters learned in the optimization process in order to restore the representation power of the network [18]. It can be deduced from Equation (5) that the width of the max pooling operator is 2; hence, the length of the time series is halved after each convolution block (i.e., $T_{l}=\frac{1}{2}T_{l-1}$).

**Figure 1:**
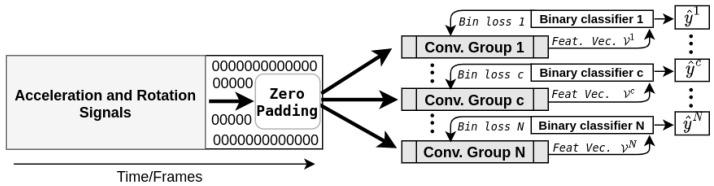
An high-level representation of the network architecture and learning mechanisms. Each class has its own convolution group that does the feature extraction and its own binary classifier.

Rather then connecting the output of the last convolution block to a classifier, as was done in previous 1D-CNN based works [9,10,11,12], the proposed method instead achieves high-level reasoning (inference and learning) by connecting to a classifier the maximum and minimum values of each dimension of each time series generated throughout the network. Equivalently, this concept can be regarded as generating a feature vector, 𝒱, by sampling elements of the different time series as such:

(7) $$V=\underset{l}{⋃}{\left\{\max_{t}\left\{x_{t}^{l,k}\right\},\;\min_{t}\left\{x_{t}^{l,k}\right\}\right\}}_{k=1}^{F_{l}}$$

and when doing so, it is important to keep a constant ordering of the elements of the feature vector. This way, the sampled values from a specific time series associated with a specific convolution filter always exploit the same connections to the classifier. For that purpose, Algorithm 1 was used in order to create the feature vectors:

As CNNs learn convolution filters that react to specific features, these maximum and minimum activation values are correlated with specific motions. To ensure that the convolution filters learn and capture discriminating dynamics for every action class, different sets of filter banks were independently learned through multiple binary classification problems and grouped convolutions. From this process, illustrated in Figure 1, N different feature vectors, ${\left\{V\right\}}_{c=1}^{N}$, were created based on the discriminating filters learned for each of the N different actions. Additionally, this process also resulted in N different binary classifiers.
**Algorithm 1:** Feature vector harvesting method.
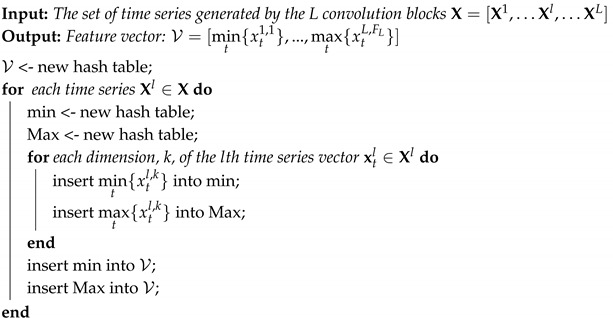


As each classifier can be taken individually with their specific set of filters in order to recognize a specific action, our method is modular. Thus, it is possible to reduce the computation and memory requirements dramatically during inference if a single or a reduced set of actions is targeted.

**Figure 2:**
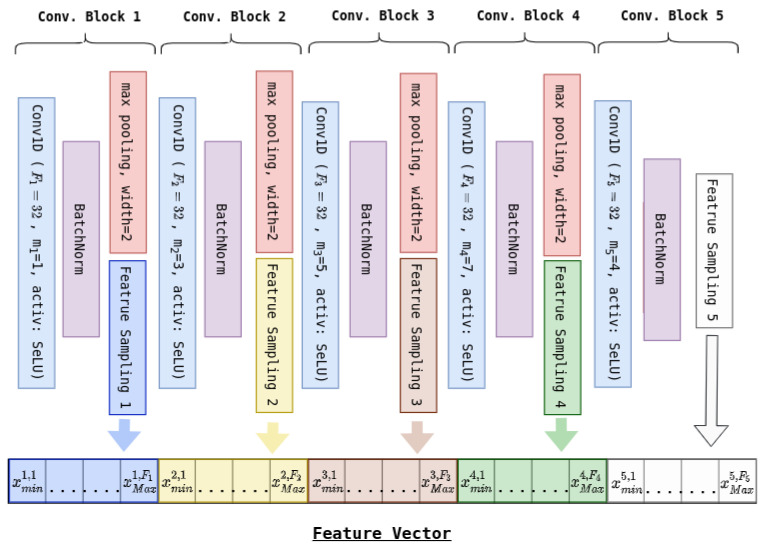
Architecture of a convolution group. For the convolution operation (in blue), $F_{l}$ and $m_{l}$ refer to the number of convolution filters ($F_{l}$) and their width along the temporal dimension ($m_{l}$) according to the conv. block to which they belong (*l*), and activ. refers to the activation function. The purple rectangle is there to illustrate the batch normalization operation applied to the convolutions’ output. From this normalized output, max pooling is applied, resulting in a new time series $X^{l}$ from which elements of the feature vector are sampled and serving as the next block’s input.

Although it is probable that performances could be improved by tailoring a specific architecture for each class, this avenue was not explored in the present work. Instead, each convolution group has the same architecture that is provided by Figure 2.

As illustrated, it consists of five consecutive blocks that are defined by the following operations:

- A multi-channel 1D convolution layer
- Batch normalization
- Max pooling
- Feature sampling

According to the proposed architecture, it is also important to observe that in the first block, the convolution kernels’ width ($m_{1}$) is set to 1; hence, instantaneous dynamics are sampled. As the information moves to subsequent blocks, longer discriminating dynamics are harvested by the coaction of both larger kernels and pooling layers compressing the temporal information while providing a certain degree of invariance towards translations and elastic distortions [19]. It is also possible to observe, in Figure 2, that each convolution layer has a total of 32 filters ($f^{l}$) from which it ensures that each feature vector (depicted at the bottom) has a total of 320 elements (32 min and 32 max for each of the 5 generated times series).

In order to train binary classifiers (see Figure 1), some relabelling had to be done. Therefore, all the examples were relabelled with respect to the different groups as 1 if the group index, c, matched the original multi-class label and 0 otherwise.

(8) $$y^{c}=\left\{\begin{array}{cc} 1, & \mathrm{if}\;y_{c}=1\\ 0, & \mathrm{otherwise} \end{array}\right.$$

This process is thus described by Equation (8), where $y^{c}$ refers to the label attributed to the examples going through the *c*th binary classifier and $y_{c}$ is the *c*th element of the original multi-class label expressed as the vector $y\in {\left\{0,1\right\}}^{N}$ such that the true class element is set to 1 and all others to 0 (one-hot-encoding).

(9) $$P_{rediction}=\arg\max_{c}{\left\{{\hat{y}}^{c}\right\}}_{c=1}^{N},\;$$

In the proposed method, predictions were made based on a one-vs.-all approach, which is depicted by Equation (9), where ${\hat{y}}^{c}$ corresponds to the probability output for the positive class of the *c*th binary classifier.

**Figure 3:**
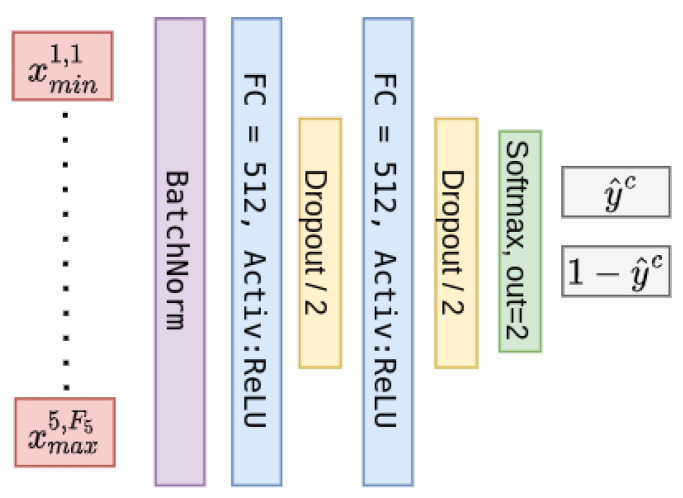
Binary classifier.

As for the convolution groups, the architectures of the binary classifiers are identical. As shown on Figure 3, the feature vector (whose elements are in red) starts by going through a batch-normalization layer (in purple) before going through two fully connected layers (in blue). Unlike the convolution modules, bias was allowed, and the chosen activation function was ReLU. Additionally, dropout layers (in yellow) randomly dropping half of the connections after both fully connected layers were inserted in order to reduce overfitting. Lastly, as part of each learning process, the classifiers’ outputs (${\hat{y}}^{c}$ and $1-{\hat{y}}^{c}$) were made “probabilistic” (positive and summing to one) using the softmax function (in green).

The cost function used during the training phase was the weighted cross-entropy, which is expressed by Equation (10). As it has been shown to be an effective way to deal with unbalanced data [20], the weights were set to be inversely proportional to the number of training examples of a specific class relative to the total number of training examples. Thus, by assigning a weight of 1 to the positive class’ examples, the weight of the negative class’ examples (label = 0) is $\frac{S_{c}}{M-S_{c}}$, where $S_{c}$ is the number of training examples of the specific class c and M is the total number of training examples.

(10) $$-y^{c}\log\left(\hat{y^{c}}\right)-\frac{S_{c}}{M-S_{c}}\left(1-y^{c}\right)\log\left(1-\hat{y^{c}}\right)$$

## 4. Temporal Analysis

With the proposed method, it is possible to localize in time each element of the feature vector used in order to make predictions and/or train the network. As a matter of fact, the functions torch.max and torch.min of the PyTorch library, used in order to create our feature vectors, return the index location of the maximum or minimum value of each row of the input tensor in a given dimension, k. Hence, this section will explain how the sampled elements of the feature vector can be associated with a confidence interval in the original time series (IMU signals) and that elements sampled from different convolution blocks report dynamics of different durations.

For the first convolution block output, this analysis is trivial. As the convolution kernels of the first block are of width one, if the returned index of the min and/or max function is t, Equation (5) indicates that this element is related to a specific dynamics that occurred either during the (2t)th or (2t+1)th time frame of the original time series (IMU signals). Unfortunately, the exact occurrence cannot be determined as pooling is not a bijective operation. Furthermore, as information gets sampled from the elements generated by deeper blocks, this uncertainty grows, but remains bounded, making it possible to determine a confidence interval for the sampled features.

**Figure 4:**
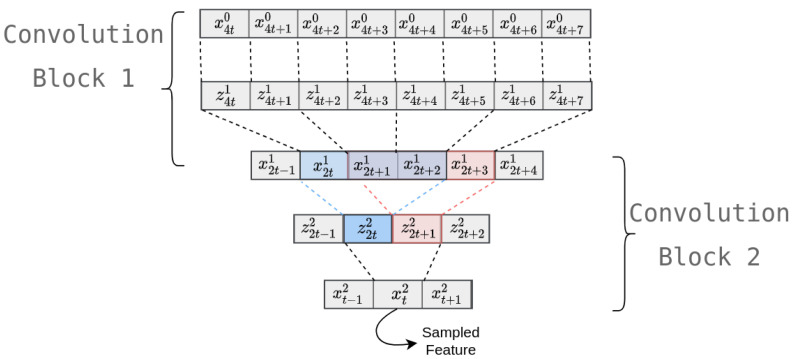
Temporal tracing of a feature of the second convolution block. The indexes correspond to those of Equation (10). The blue and red rectangles express the two different positions of the same convolution kernel of the second convolution block.

In order to illustrate this process, let us take a look at Figure 4, which takes the previous example one convolution block deeper. For both convolution blocks, the first line refers to the block’s input; the following is the result of the convolution layer; and the third is the result of the pooling layer. In order to simplify the analysis, the time step vectors only have one dimension, and batch normalization is omitted as it has no influence on the temporal traceability. As the second block’s convolution filter has a width of three (i.e., $m_{2}=3$), if a feature is sampled from the second time series, $X^{2}$, with a relative position index of t, the corresponding dynamic consequently occurred between the (4t)th and the (4t+7)th time steps of the original signal.

Generally speaking, knowing that the stride of the convolutions are set to one and the max pooling width is two, it is possible to determine the length of a certain feature based on the convolution block from which it was sampled.

Defining $D^{l}$, $m_{l}$ and $E^{l}$ as the duration of the features, the width of the convolution filters and the temporal uncertainty caused by max pooling of the *l*th convolution block, it is possible to inductively calculate the duration of each convolution block feature using the following set of equations:

(11) $$\begin{array}{cc} \; & E^{l}=2^{l-1}\\ \; & D^{1}=1\\ \; & D^{l}=D^{l-1}+E^{l-1}+\left(m_{l}-1\right)\times 2^{l-1} \end{array}$$

Consequently, the confidence interval, I, during which the dynamic of a certain feature occurred, can be expressed as:

(12) $$I_{t}^{l}\in [t\times 2^{l},t\times 2^{l}+D^{l}+E^{l}[$$

Table 1 gives the relative duration, D, the relative uncertainty, E (both with respect to the original time step), and the real duration (knowing that the original signal was acquired at 30 Hz) based on the layer from which they were sampled and the width of the convolution filters specified by the proposed architecture.

## 5. Experiments

Using the publicly available UTD-MHAD dataset [21], which proposes a total of 27 different actions performed 4 times by eight different subjects (4 males and 4 females), this section will first demonstrate how our hierarchical framework makes our method robust towards zero-padding and that it is possible to train and infer on sequences of different lengths without impacting the performances. Then, we will assess the performances and compare them with state-of-the-art methods. Finally, we will conduct an analysis on the complexity of the method and see how it compares with some well-known CNN architectures.

**Figure 5:**
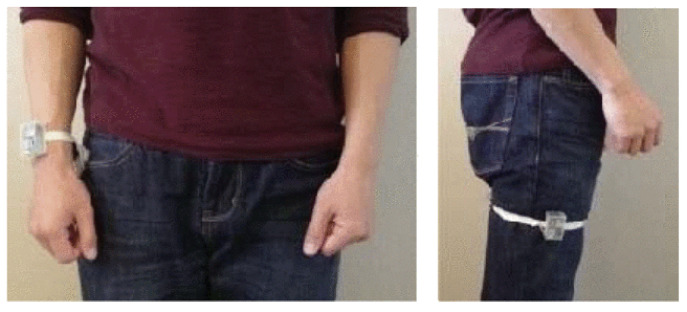
Wearable inertial sensor placements [21].

Although it provides RGB, depth and skeleton streams, our experiments were conducted only on the inertial sensor signals, which were acquired from a single IMU worn on the right wrist for Actions 1 through 21 and on the right tight for Actions 22 through 27, as displayed in Figure 5. For all our experiments, the evaluation protocol suggested in the original UTD-MHAD paper [21] was followed, where odd subjects are for training and evens for evaluation, except in Section 5.2, which will additionally provide an assessment of the performances through a leave-one-out k-fold cross-validation protocol. In all cases, the experiments were performed using the PyTorch deep learning framework with stochastic gradient descent as the optimizer and a batch size of 16. The learning rate was initialized at 0.01 and decayed 1% per epoch. Weights were initialized using the Xavier method, and the bias of the classifier was initialized using a Gaussian distribution of mean 0 and variance 1.

### 5.1. Robustness against Zero-Padding and Flexibility towards the Input’s Length

**Figure 6:**
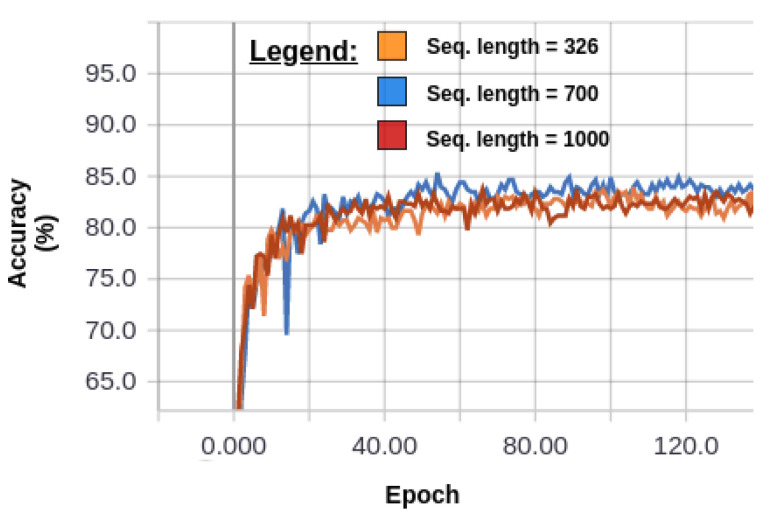
Learning curves assessing the accuracy obtained on the test set (*Y* axis) relative to the number of training epochs (*X* axis) for sequences (Seq.) zero-padded up to different extent.

The idea behind our feature sampling method was to create a feature vector based on information sampled from multiple local signals instead of encoding the whole sequence into a feature vector. Hence, meaningless and/or redundant information such as zero-padding does not affect the feature vector, nor the classification performances. In order to validate this hypothesis, signals were extended up to various lengths with zero-padding. More precisely, training using sequences normalized up to 326, 700 and 1000 frames was conducted, for which the results are reported by Figure 6.

As we can see, the performances are rather steady around 83% regardless of the amount of zero-padding added to the original sequence. This is an interesting result as Imran and Raman [9] previously conducted a 4-fold cross-validation analysis using only the subjects of the training set and observed that using the conventional CNN approach, where only the information encoded in the last layer is provided to the classifier, zero-padding the IMU signals of UTD-MHAD dataset caused the accuracy to decay by nearly 7%. More precisely, they transitioned from randomly sampled sequences of 107 time frames (which corresponded to the duration of the shortest action) to sequence zero-padded up to 326 time frames (which corresponded to the longest action). Intermediate lengths were also tested, where shorter sequences were zero-padded, while longer ones were randomly sampled. As observed on Table 2, their accuracy kept decreasing as the amount of zero-padding increased.

Moreover, it was also observed that with our hierarchical method, it was possible to train the model using sequences of a certain length and proceed to classify sequences of a different length, which is impossible with the other CNN approaches. As a matter of fact, the parameters learned at the 100th epoch of the model trained with the sequence extended to 700 time frames (see Figure 6) resulted in an accuracy of 84.88%, which was also reproduced regardless of the testing examples being zero-padded up to 326 or 1000 time frames.

### 5.2. Performances

**Figure 7:**
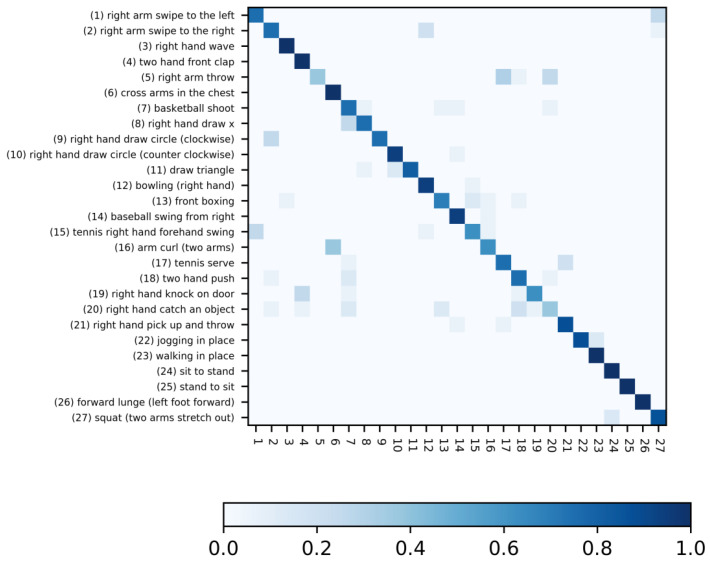
Confusion matrix obtained using the one-vs.-all discriminating approach.

With our method, we obtained a maximal accuracy score of 85.35%, which resulted in the confusion matrix provided by Figure 7. As we can see, the performances were better on actions for which the inertial sensor was placed on the thigh rather than on the wrist.

In order to compare our result, we built Table 3, which indicates the accuracy of previous methods and the modality that they used in order to achieve it. Furthermore, we also report the performances on the NTU-RGB-D dataset for the methods that validated their approach on it using the cross-subject/cross-view evaluation protocol (CS/CV), as it is a well-established benchmark for HAR. As we can see, our method is very competitive as it outperformed the current state-of-the-art using inertial data before it benefited from the data augmentation, which, in our case, was not implemented. Another important observation that can be made by taking a closer look at Table 3 is that the best overall performance was achieved by Imran and Raman [9] through a multi-stream fusion approach using inertial signals. Moreover, in the conclusion of their work, Imran and Raman argued that it was not only the complementarity between the different modalities that helped them achieve these results, but also the complementarity between the architecture treating the different data streams, thus further stressing the importance of 1D-CNN architectures.

In order to compare the performances of our method against the one proposed by Wei et al. [28] that uses a 2D-CNN architecture applied to images generated from the IMU’s signals, we also proceeded to the evaluation of the performances using a leave-one-out 8-fold cross-validation, for which the results are summarized by Table 4.

Computing the mean value of all these runs, we get a value of 90.8%, which is slightly better than the 90.3% obtained by Wei et al. [28].

### 5.3. Computational Complexity Analysis

As stated earlier, one of the greatest challenges currently faced by HAR algorithms is their portability to limited resource electronics; hence, we proceeded to do an analysis of the computational complexity and the number of parameters, which ended up demonstrating that our approach could be very easily integrated in limited resource electronics.

**Figure 8:**
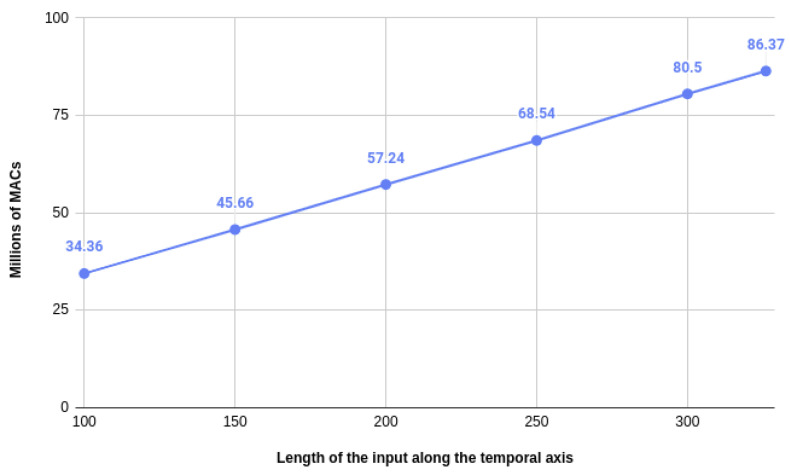
Learning curves assessing the accuracy obtained on the test set (*Y* axis) relative to the number of training epochs (*X* axis) for different amounts of zero-padding. MAC, multiply and accumulate.

As a matter of fact, the number of operations needed by our model is directly correlated to the normalized length of the input sequence. As we can see in Figure 8, the relation is linear and is equal to 86.37 million multiply and accumulate (MAC) operations when the normalized length is equal to 326.

**Figure 9:**
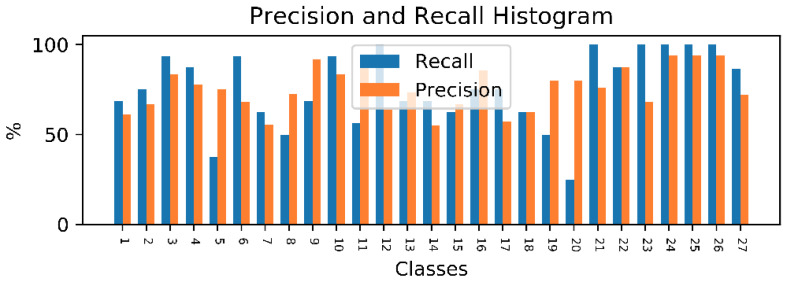
Histogram reporting the precision and recall score for each class.

Moreover, as our method is modular, the performance of each binary classifier can be studied independently. For that purpose, the precision and recall scores for each class are given by Figure 9.

**Figure 10:**
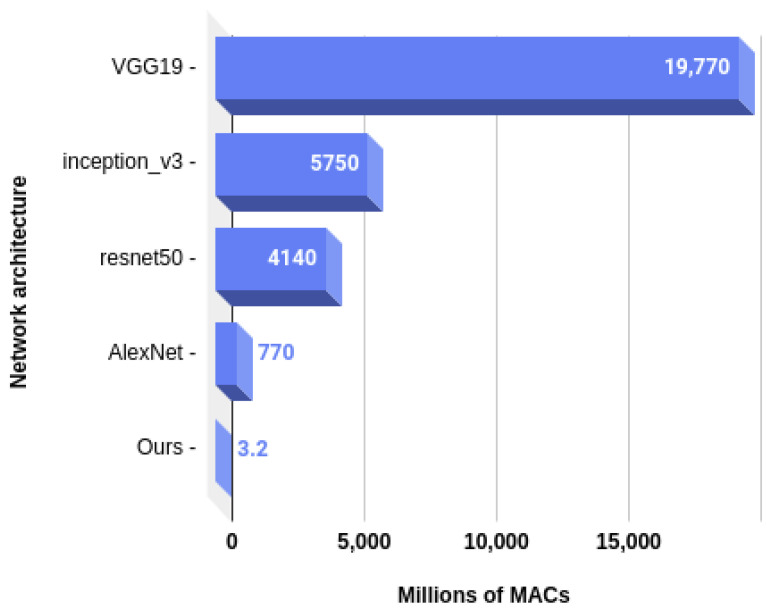
Number of computations needed by our model compared to popular CNN architectures.

**Figure 11:**
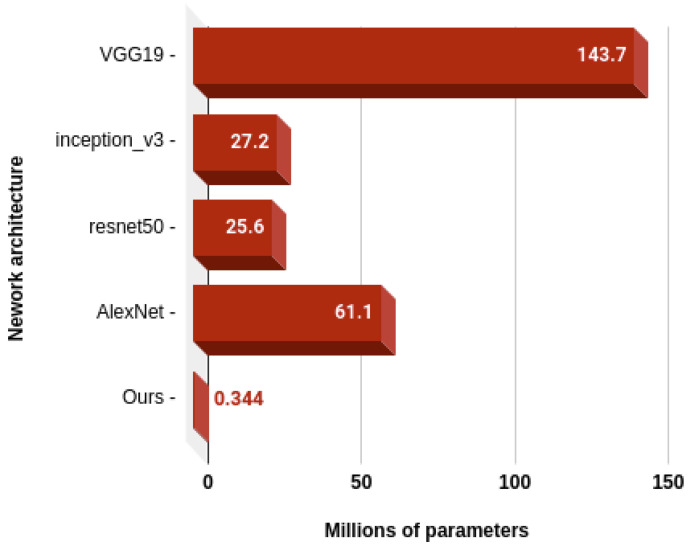
Number of parameters needed by our model compared to popular CNN architectures.

Therefore, the number of operations and parameters can be divided by 27 in order to be integrated into a simple device only recognizing one action. In that case, the number of parameters would only be 343,830, which only takes about 1.4 MB of memory if the parameters are represented with single precision floating-point numbers, as was the case in our experiments. In the case of the number of operations, it comes down to about 3.2 million MACs. As MACs account for two floating point operations (one multiplication and one addition), if a processing unit has a throughput of 1 operation per clock cycle, a processing speed of only 3.2 MHz would be necessary in order to run our model under 1 s. As many inexpensive microcontrollers offer sufficient performances, this proves that our method could easily be embedded into smart devices. Finally, comparing our method with some of the most popular CNN architectures regarding the number of computations, on Figure 10 and the number of parameters on Figure 11, we can appreciate once more how hardware-friendly our method really is.

## 6. Discussion

In this paper, we presented a new framework based on a 1D-CNN architecture in order to perform action recognition on signals generated by IMUs. Although less accurate than methods using visual streams, this approach still has its advantages as it provides more privacy and can be used anywhere, as long as wearing the acquisition device does not become too inconvenient. Moreover, we demonstrated that our method is computationally inexpensive enough to be integrated into embedded devices. On another note, as the latent representation can be viewed as an organized set of elements assessing the presence of specific sub-events, the proposed method works in a hierarchical way. Yet, the temporal relationships between these sub-events are not captured implicitly or explicitly; thus, we believe that the proposed approach could help address the unsolved problem [29] of high-level action recognition with machine learning based methods as the difficulty that previous algorithms faced with this type of actions lies in the fact that they have important temporal variations between the sub-events composing them. This hierarchical approach also allowed us to infer on times series extended up to various lengths without compromising the accuracy.

Finally, we also demonstrated that the element of the latent representation can be traced in time, both in location and duration. In future work, we would like to investigate this ability further as we believe that it offers more transparency to the inner workings of the model by providing a way to interpret them. For example, this could be done by assessing if specific kernels are in fact reacting or not to discriminating movements based on the movements occurring in their confidence interval. On top of opening a window into the black-box of deep-learning algorithms, we believe that this approach could help reduce over-fitting and propose better architectures, tailored to specific actions, thus improving the overall performances.

## Figures and Tables

**Table 1:** Characterisation of the uncertainty (E) and relative duration (D) of the sampled features based on the networks hyper-parameters (l) and (m).

Conv. Block (l)	Kernel Width (m)	Relative Duration (D)	Relative Uncertainty (E)	Feature Duration
1	1	1	1	0.033 s
2	3	6	2	0.2 s
3	5	22	4	0.73 s
4	7	70	8	2.33 s
5	4	118	16	3.93 s

**Table 2:** Mean accuracy of the k-fold cross-validation analysis produced by Imran et al. [9] with respect to zero-padding on their 5 layer 1D-CNN.

**Normalized Sequence Length**	107	180	250	326
**Max. Accuracy**	83.99%	80.05%	78.42%	77.04%

**Table 3:** Comparison with the literature. CS/CV, cross-subject/cross-view.

Buffer Size	Modality	UTD-MHAD	NTU-RGB-D (CS/CV)
Chen et al. [21]	Inertial	67.20%	
Imran et al. [9] (w/o data augmentation)	Inertial	83.02%	
Imran et al. [9] (with data augmentation)	Inertial	86.51%	
Hussein et al. [22]	Skeleton	85.58%	
Wang et al. [23]	Skeleton	87.90%	76.32%/81.08%
Hou et al. [24]	Skeleton	86.97%	
Imran et al. [9]	Skeleton	93.48%	
Li et al. [25]	Skeleton	95.58%	82.96%/90.12%
Chen et al. [21]	Depth + inertial	79.10%	
Wang et al. [26]	Depth + skeleton	89.04%	
El Madany et al. [27]	Depth + Inertial + Skeleton	93.26%	
Imran et al. [9]	RGB + inertial + skeleton	97.91%	
**Proposed method**	Inertial	85.35%	

**Table 4:** k-fold validation on UTD-MHAD.

Test Subject	1	2	3	4	5	6	7	8
**Accuracy**	96.3%	89.8%	88.0%	90.7%	94.4%	91.6%	90.7%	85.1%

## Abbreviations

The following abbreviations are used in this manuscript:

**1D-CNN**	One-dimensional convolution network
**HAR**	Human action recognition
**IMU**	Inertial measurement unit
**IoT**	Internet of Things
**k-NN**	k-nearest neighbour
**LSTM**	Long short-term memory
**MLP**	Multi-layer-perceptron
**MAC**	Multiply–Accumulate operation
**ReLU**	Rectified Linear Unit
**RNN**	Recurrent neural network
**SeLU**	Scaled exponential linear unit
**SVM**	Support vector machine
**UTD-MHAD**	University of Texas at Dallas-Multimodal Human Action Dataset
